# How Pet Attachment Is Associated with Human–Pet Co-Fashion Purchase Intention: The Roles of Relational Identity and Fashion Involvement

**DOI:** 10.3390/bs16091544

**Published:** 2026-09-01

**Authors:** Yuechun Ding, Lixian Liu

**Affiliations:** 1 School of Art and Design, Zhejiang Sci-Tech University, Hangzhou 310018, China; 2025111205002@mails.zstu.edu.cn; 2 School of Fashion Design and Engineering, Zhejiang Sci-Tech University, Hangzhou 310018, China; 3 Silk and Fashion Culture Research Center of Zhejiang Province, Zhejiang Sci-Tech University, Hangzhou 310018, China

**Keywords:** pet attachment, human–pet relational identity, human–pet co-fashion, pet fashion product consumption, fashion involvement

## Abstract

As pets increasingly function as family members, emotional companions and resources for identity expression, pet-related fashion consumption has expanded from pet clothing to human–pet co-fashion, in which owners and pets present a coordinated style relationship. Yet the mechanism linking pet attachment to co-fashion purchase intention, and the boundary condition shaping this link remain unclear. Drawing on survey data from 565 consumers with current or prior pet ownership experience, this study examines pet attachment, human–pet relational identity, fashion involvement, pet fashion product purchase intention and human–pet co-fashion purchase intention through mediation and moderation analyses. Pet attachment was positively associated with both purchase intentions, and human–pet relational identity partially mediated these associations. Pet attachment and relational identity were more strongly associated with co-fashion purchase intention than with pet fashion product purchase intention. Fashion involvement negatively moderated the relationship between relational identity and both purchase intentions, indicating that the positive association weakened as fashion involvement increased. By conceptualising human–pet co-fashion as co-consumption that externalises relational identity, this study clarifies how human–pet relationships are expressed through fashion consumption.

## 1. Introduction

Pets are increasingly embedded in consumers’ emotional lives and identity practices. They have become important vehicles for self-expression and relationship construction (Albert & Bulcroft, 1988; Dwyer et al., 2006; Beck & Madresh, 2008; Xia et al., 2023). This shift is also evident in China. In 2025, urban China had approximately 126 million pet cats and dogs. This population included about 72.89 million cats and 53.43 million dogs. The associated consumer market exceeded RMB 312.6 billion (Petdata, 2026). Cats and dogs constitute the principal companion-animal categories in China’s mainstream pet market and in the consumption context relevant to this study. Other companion animals occupy a much smaller position in this market context. Yet pet-keeping practices in China are not homogeneous. They vary with socioeconomic conditions, urban–rural environments, and generational differences. Wu et al. (2024) found that pet ownership was significantly associated with socioeconomic conditions at the individual, household, and regional levels. Urban residents were also more likely to own pets than rural residents. Su and Martens (2017) found that younger Chinese people held more positive attitudes towards animals than middle-aged and older groups. Yin et al. (2025) further showed that personal attitudes and subjective norms significantly shaped pet-ownership intentions among young Chinese people. These differences indicate that human–animal relationships in China should not be treated as socially or geographically homogeneous.

As pets acquire greater emotional and relational significance, pet consumption is expanding beyond food, healthcare, and routine care. It now encompasses more symbolic domains, including clothing, photography, and social display (Hill et al., 2008; D’Souza et al., 2023; Liu et al., 2024). Pet fashion is a prominent manifestation of this shift. Previous research shows that pet attachment can encourage purchases of pet apparel (Apaolaza et al., 2022). Pet-related consumption can also contribute to construction of owners’ identities and the meaning of human–pet relationships (Xia et al., 2023). Existing studies and market statistics on pet fashion primarily examine cats and dogs, with dogs receiving particular attention (Apaolaza et al., 2022; Xia et al., 2023). Accordingly, although this article retains the broader term “pet”, its substantive discussion of pet fashion is primarily situated in the context of cats and dogs. This scope is also consistent with the fact that wearable pet fashion products and owner–pet coordinated apparel are discussed in the existing literature mainly in relation to these companion animals.

This increasingly symbolic market has given rise to a practice distinct from conventional purchases of pet apparel. Recent research has begun to document owner–pet matching outfits, in which owners coordinate their appearance with that of their pets as a form of expressive consumption (Chen et al., 2026). Consumers may coordinate their own and their pets’ clothing, colours, patterns, or styles to present themselves as a visually coherent unit. We term this practice “human–pet co-fashion”. This conceptualisation is consistent with research suggesting that owners and pets may constitute a joint consumption unit (Kylkilahti et al., 2016) and with evidence from coordinated human dress showing that matching appearance can communicate relationship status and shared identity (Park, 2013). Conventional pet fashion consumption has primarily focused on apparel purchased for the pet itself (Apaolaza et al., 2022). By contrast, human–pet co-fashion incorporates both owner and pet into a shared visual expression. The focal question therefore shifts from “What does the pet wear?” to “How are the owner and pet presented together?” Human–pet co-fashion thus involves not only pet-apparel consumption but also the visual expression of the human–pet relationship.

Although recent research has begun to examine matching owner–pet apparel, current explanations concentrate on proximal determinants of consumer decisions. Chen et al. (2026), for example, explain purchase intentions through attitudes, subjective norms, perceived behavioural control, and aesthetic risk. Such research identifies the cognitive conditions under which consumers become more willing to buy matching apparel. It provides less insight into a more fundamental question. Why do consumers move beyond buying clothing for pets and include themselves in a shared fashion expression? Existing research has therefore not fully explained how an emotional human–pet bond becomes relationship-oriented fashion consumption.

We argue that this process involves three levels: emotional attachment, relational identity, and fashion-related characteristics. First, pet attachment can explain why owners continually invest resources in their pets. However, a strong emotional bond does not necessarily motivate owners to express the relationship through coordinated dress. Second, this bond may become a relational identity when owners incorporate the human–pet relationship into their self-definition. We refer to this construct as human–pet relational identity. Human–pet co-fashion directly expresses the owner–pet relationship as a unit, unlike conventional pet fashion purchases. It may therefore be more directly driven by relational identity. Finally, human–pet co-fashion remains a fashion practice. Whether relational identity translates into purchase intention may consequently depend on the consumer’s level of fashion involvement.

Building on this logic, we develop a framework linking pet attachment, human–pet relational identity, fashion involvement, and two forms of purchase intention. We examine intentions to purchase pet fashion products and intentions to purchase human–pet co-fashion products. Comparing these outcomes distinguishes pet-oriented consumption from consumption oriented towards the human–pet relationship. It also reveals how emotional bonds translate through relational identity into distinct forms of fashion consumption. Moreover, this comparison identifies the individual characteristics that condition this process.

This study makes three theoretical contributions. First, we introduce and define human–pet co-fashion as a relationship-oriented fashion-consumption practice. This practice visually expresses the human–pet relationship by coordinating the appearances of the owner and pet. The concept distinguishes such consumption from conventional purchases of pet fashion products. Second, we introduce human–pet relational identity to explain how pet attachment becomes associated with relationship-oriented consumption through relational self-definition. This account extends research that explains pet consumption primarily through anthropomorphism, the extended self, and self-expansion. Third, we incorporate fashion involvement into the framework. This approach establishes human–pet co-fashion as both relational consumption and fashion consumption. It also identifies when relational identity is more or less likely to translate into purchase intention. Practically, the findings can help pet fashion brands recognise consumers’ needs for relational expression. Brands can then develop coordinated product systems, contextualised communications, and more refined segmentation strategies.

## 2. Literature Review and Hypothesis Development

### 2.1. Human–Pet Co-Fashion: A Relationship-Oriented Fashion Practice

Fashion is more than a choice of clothing. It is a social practice through which appearance communicates aesthetic preferences, social distinction, and identity (Simmel, 1957; Roach-Higgins & Eicher, 1992). Human–pet co-fashion extends these functions while introducing distinctive visual and relational properties through its cross-species, dual-subject structure. Owners coordinate their clothing, colours, and styles with those of their pets. This coordination organises two separate appearances into a coherent visual image. Co-fashion does not require identical clothing. Instead, it incorporates both participants into a shared style system through deliberate visual coordination. This unconventional combination is more visually salient than individual dress and may therefore attract greater attention (Itti & Koch, 2001).

Such visual distinctiveness may also enhance social visibility. Pets can facilitate interactions among strangers in public spaces (McNicholas & Collis, 2000). Coordinated appearances can also communicate relationship status and shared identity to others (Park, 2013). Human–pet co-fashion is therefore more than an aesthetic choice. Through recognisable visual symbols, it can bring an otherwise private human–pet relationship into the public sphere.

The defining feature of human–pet co-fashion is that its expressive unit shifts from “I” to “we”. Fashion research has long examined how clothing expresses individual identity (Roach-Higgins & Eicher, 1992). Coordinated dress can additionally communicate relational identity (Park, 2013). In human–pet settings, pets participate in consumption through bodily responses and behavioural preferences. However, they cannot negotiate the symbolic meaning of a shared appearance as human partners do (Kylkilahti et al., 2016; Vänskä, 2016). Human–pet co-fashion is therefore best understood as a relational fashion practice initiated by the owner but shaped by the pet’s behavioural participation. This practice further increases the social visibility of human–pet relationships. Pets are increasingly regarded as family members and relational partners. Nevertheless, these cross-species relationships remain socially and institutionally distinct from conventional marital and kinship relations (Charles, 2014; Pallotta, 2019). By coordinating appearances, owners can translate an abstract human–pet relationship into a more readily recognisable visual symbol.

Accordingly, we define human–pet co-fashion as a fashion-consumption practice in which owners deliberately coordinate their appearance with that of their pets. Such coordination may involve clothing styles, colour schemes, or visual symbols. In public or private settings, the practice presents the owner and pet as a relational unit.

### 2.2. Relational Self Theory and Human–Pet Relational Identity

Traditional research on the self has primarily understood it through internal attributes, including personal traits, abilities, and attitudes. A relational perspective further proposes that relationships with significant others can organise the self (Andersen & Chen, 2002; Brewer & Gardner, 1996). Cross et al. (2000) introduced the relational-interdependent self-construal (RISC) concept. RISC describes a general tendency to define the self through close relationships. Individuals with higher RISC more readily incorporate representations of significant others into self-relevant information processing. This tendency shapes relational memory, self-evaluation, and behavioural judgement (Cross & Morris, 2003; Cross et al., 2002). Behaviour may therefore arise from an independent personal identity or from how individuals understand themselves within relationships.

Extending this perspective to human–pet contexts requires the establishment of whether pets can become relational others with self-relevant meaning. Some owners regard pets as emotionally significant family members, companions, or even kin. They also form enduring, intimate bonds with them (Greenebaum, 2004; Charles, 2014). Moreover, owners do not unilaterally determine the meaning of human–pet relationships. Haraway’s (2003, 2008) companion-species perspective emphasises that relationships emerge through sustained human–animal interactions. Consumer research similarly shows that pets shape consumption practices through their behaviour, preferences, and bodily responses (Cheetham & McEachern, 2013; Vänskä, 2016; Kylkilahti et al., 2016). Pets cannot engage in symbolic negotiation as fully as human partners. Nonetheless, these interactions allow pets to become relational others with enduring significance.

However, RISC is a trait-like orientation that captures how individuals generally organise the self through close relationships. It is not anchored in a particular relationship. We therefore shift the analytical focus from “the pet entering the self” to “the relationship entering the self”. The central issue is not whether the pet becomes part of the self. Rather, it is whether the relationship with a particular pet becomes a basis for self-understanding. We accordingly introduce human–pet relational identity. We define it as the extent to which owners incorporate their relationship with a particular pet into their self-definition.

### 2.3. Pet Attachment and Fashion Consumption

The effects of pet attachment on consumer behaviour have been demonstrated across several contexts. Stronger human–pet emotional bonds are closely associated with owners spending on pet care, healthcare, and related products (Shore et al., 2005; Brockman et al., 2008; Boya et al., 2012; Jyrinki, 2012; D’Souza et al., 2023). In pet-apparel settings, Apaolaza et al. (2022) found that pet attachment significantly increased intentions to purchase fashionable pet clothing. Pet-related consumption can therefore constitute a material practice through which owners express and sustain their emotional bonds with pets.

This effect may first appear in purchases of conventional pet fashion products. Pet apparel can serve practical purposes while also functioning as a symbolic means through which owners express care, attachment and identity-related meanings associated with their pets (D’Souza et al., 2023). Previous research further shows that stronger pet attachment is positively associated with consumers’ intention to purchase fashion pet clothing (Apaolaza et al., 2022). Accordingly, stronger pet attachment may correspond with greater intention to purchase pet fashion products.

Human–pet co-fashion additionally requires owners to incorporate themselves into the consumption display. Unlike choosing apparel only for a pet, co-fashion directly presents the human–pet relationship. Research on relationship visibility shows that people use external symbols to incorporate important relationships into the self-images communicated to others (Emery et al., 2014). Coordinated dress can likewise express relationship status and shared identity (Park, 2013). Strong pet attachment may therefore encourage both pet-oriented spending and the presentation of the relationship through a shared appearance.

The two forms of consumption consequently have different expressive foci. Purchases of pet fashion products focus primarily on the pet, whereas human–pet co-fashion directly presents the relationship. Because co-fashion is more closely tied to relational expression, pet attachment should have a stronger positive association with co-fashion purchase intention. We therefore propose:

**H1a.**  *Pet attachment is positively associated with pet fashion product purchase intention.*

**H1b.**  *Pet attachment is positively associated with human–pet co-fashion purchase intention.*

**H1c.**  *The positive association between pet attachment and human–pet co-fashion purchase intention is stronger than that between pet attachment and pet fashion product purchase intention.*

### 2.4. The Mediating Role and Differential Effects of Human–Pet Relational Identity

As discussed in Section 2.2, stronger pet attachment may increase the self-relevance of the human–pet relationship. It may also embed that relationship more deeply in the owner’s self-understanding. Previous studies show that pets can participate in consumer identity construction. Pet attachment is also closely associated with incorporating pets into the self-structure (Jyrinki, 2012; Apaolaza et al., 2022; D’Souza et al., 2023). Owners with stronger pet attachment may therefore be more likely to treat the relationship as central to their self-definition.

Once a relational identity forms, consumers often prefer consumption forms that align with and express their self-definition. Self-congruity research shows that alignment between the self-concept and a consumption object’s meaning can promote favourable consumer responses (Aguirre-Rodriguez et al., 2012). In human–pet fashion, conventional pet fashion primarily presents the pet. Human–pet co-fashion instead presents a relational “we” through coordinated dress. Similar forms of coordinated clothing can communicate relationship status and shared identity (Park, 2013). Human–pet relational identity should therefore be positively associated with both purchase intentions. However, its association with human–pet co-fashion purchase intention should be stronger.

Human–pet relational identity may thus provide an indirect pathway between pet attachment and purchase intention. Stronger attachment is associated with a stronger human–pet relational identity, which is subsequently associated with fashion-consumption intentions. Human–pet co-fashion is more directly congruent with relational identity as an expressive form. This indirect association should therefore be stronger for human–pet co-fashion. We propose:

**H2.**  *Pet attachment is positively associated with human–pet relational identity.*

**H3a.**  *Human–pet relational identity is positively associated with pet fashion product purchase intention.*

**H3b.**  *Human–pet relational identity is positively associated with human–pet co-fashion purchase intention.*

**H3c.**  *The positive association between human–pet relational identity and human–pet co-fashion purchase intention is stronger than that between human–pet relational identity and pet fashion product purchase intention.*

**H4a.**  *Human–pet relational identity mediates the relationship between pet attachment and pet fashion product purchase intention.*

**H4b.**  *Human–pet relational identity mediates the relationship between pet attachment and human–pet co-fashion purchase intention.*

### 2.5. The Moderating Role of Fashion Involvement

Expressing human–pet relational identity through co-fashion requires more than relational motivation. Consumers must also be willing to translate relational meaning into clothing, colour, and style. Fashion involvement refers to the importance and relevance individuals assign to clothing, fashion trends, styling, and aesthetic expression. It is associated with attention to fashion information, styling knowledge, aesthetic experience, and identity communication (Zaichkowsky, 1985; Mittal & Lee, 1989; Fairhurst et al., 1989; Flynn & Goldsmith, 1993; O’Cass, 2000, 2004). Human–pet co-fashion therefore combines relational motivation with interest-based fashion motivation.

Their interaction may take three theoretically plausible forms. First, fashion involvement may strengthen the association. Highly involved consumers possess greater fashion sensitivity and styling knowledge, which can help translate relational meaning into concrete choices (Tigert et al., 1976; Fairhurst et al., 1989). Second, relational identity and fashion involvement may operate independently. Interests, values, and identities can jointly support behaviour without necessarily altering one another’s effects (Deci & Ryan, 2000). Third, fashion involvement may reduce the marginal contribution of relational identity. This may occur when pet apparel and coordinated styling already possess strong intrinsic fashion appeal (Kahneman, 1973; Payne et al., 1993; Shiv & Fedorikhin, 1999).

Human–pet co-fashion remains an emerging consumer phenomenon, and all three patterns are theoretically plausible. We therefore cannot confidently predict the direction of the interaction a priori. Instead, we treat fashion involvement as an exploratory boundary condition for both forms of purchase intention:

**H5a.**  *Fashion involvement moderates the relationship between human–pet relational identity and pet fashion product purchase intention.*

**H5b.**  *Fashion involvement moderates the relationship between human–pet relational identity and human–pet co-fashion purchase intention.*

**Figure 1:**
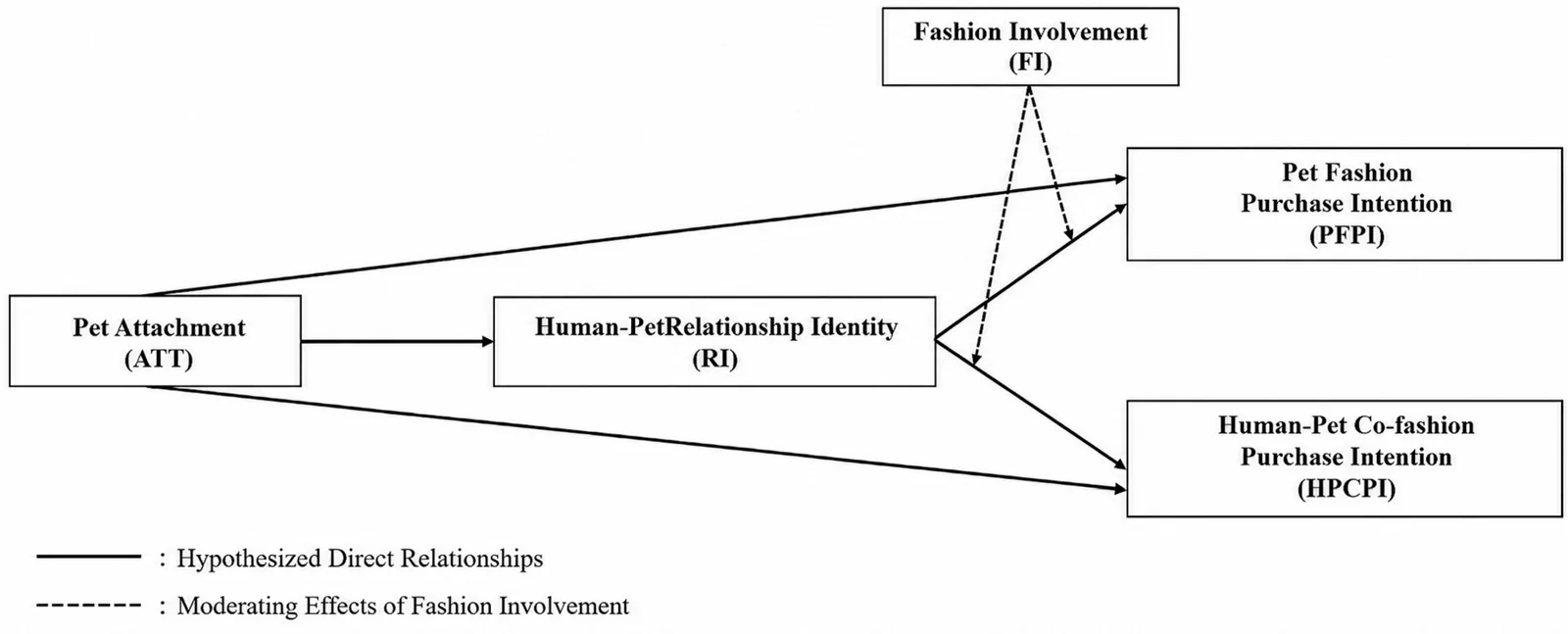
Theoretical model.

The proposed theoretical model is presented in Figure 1.

## 3. Materials and Methods

### 3.1. Data Collection and Participants

Data were collected through an online questionnaire administered via Credamo, a Chinese online research platform for questionnaires and participant recruitment. The platform supports targeted sample screening, questionnaire quality control and paid participation management, making it suitable for consumer behaviour research. The questionnaire was open to consumers in mainland China, and participants were recruited from different provinces and cities to improve geographic diversity.

To ensure research ethics and data quality, the first page of the questionnaire explained the study purpose, response requirements, anonymity principle, intended use of the data, and voluntary nature of participation. Participants could proceed to the formal questionnaire only after reading this information and providing informed consent. All questionnaire data were used solely for academic research. No personally identifiable information, such as names, identity card numbers, or contact details, was collected.

The research procedure followed academic ethical standards and the basic principles of the Declaration of Helsinki. Participants received a small monetary reward after completing the questionnaire.

Because this study focuses on human–pet relationships and their role in human–pet fashion consumption, the questionnaire began with a screening question on pet ownership experience. Participants who currently own pets were instructed to answer based on their actual relationship with their current pets and their related consumption attitudes. Participants who do not currently own pets but had prior pet ownership experience were instructed to answer based on their past experience. Participants who had never owned pets and lacked relevant human–pet interaction experience were excluded from the formal analysis. Pet species was not recorded as a separate demographic variable; the analysis therefore cannot compare dog, cat and other companion-animal owners.

Before the formal items, the questionnaire also provided a brief explanation of “human–pet co-fashion.” It was described as a coordinated, matching, or mutually echoing fashion image created through the coordination of clothing, accessories, colours, or overall style between owners and pets.

A total of 665 questionnaires were collected. The data were screened in two stages to ensure that the final sample matched the research context and met basic quality requirements. First, respondents who had never owned pets were excluded, as they lacked direct human–pet interaction experience relevant to the study. Second, attention-check items were used to identify inattentive reading, careless responding and other clear signs of invalid answers. After these screening procedures, 565 valid responses remained for analysis, corresponding to a valid response rate of 84.96%. The sample profile is summarised in Table 1.

Overall, the sample consisted primarily of consumers with current or prior pet ownership experience. It therefore matched the research context of human–pet co-fashion consumption and pet fashion consumption. However, because respondents were recruited through online channels, the sample may disproportionately represent consumers who are more active in digital environments, and the findings should therefore be interpreted within this sampling context.

### 3.2. Construct Measures

The core variables in this study were pet attachment, human–pet relational identity, pet fashion product purchase intention, human–pet co-fashion purchase intention and fashion involvement.

Except for fashion involvement, all constructs were measured using seven-point Likert scales, with 1 indicating “strongly disagree” and 7 indicating “strongly agree.” Fashion involvement was measured using a seven-point semantic differential scale to capture respondents’ evaluative orientation towards fashion.

The measurement items were adapted from established scales and revised to fit the human–pet fashion context. Item scores were averaged to form composite scores, and reverse-coded fashion-involvement items were recoded so that higher values consistently indicated stronger involvement. The purchase-intention measures referred to pet clothing and coordinated owner–pet clothing at a general category level; they did not specify a particular garment category, design style, or wearing occasion. Accordingly, product- and context-specific differences were not controlled in the present survey. These measures capture self-reported purchase intention rather than actual purchase, wearing frequency, or use behaviour. The full items, reliability results, and literature sources are reported in Table 2.

## 4. Results

### 4.1. Reliability, Validity, and Common Method Bias Tests

Before testing the hypotheses, this study examined the reliability, convergent validity, discriminant validity, and potential common method bias of the core constructs. The core constructs included pet attachment (ATT), relational identity (RI), human–pet co-fashion purchase intention (HPCPI), pet fashion product purchase intention (PFPI), and fashion involvement (FI). The results are reported in Table 3.

Cronbach’s alpha values ranged from 0.797 to 0.904, exceeding the recommended threshold of 0.70 and indicating acceptable internal consistency. Convergent validity was assessed using composite reliability (CR), average variance extracted (AVE), and standardised factor loadings. CR values ranged from 0.802 to 0.904, all exceeding 0.70. The AVE values of PFPI, HPCPI, and FI exceeded 0.50. The AVE values of ATT and RI were 0.373 and 0.464, respectively, which were slightly below the conventional 0.50 threshold. However, their CR values were 0.805 and 0.810, and their Cronbach’s alpha values were 0.807 and 0.802, respectively. In addition, all standardised factor loadings were significant and exceeded 0.50. Specifically, the standardised loadings ranged from 0.537 to 0.704 for ATT and from 0.573 to 0.831 for RI. Therefore, the measurement quality of these constructs was considered acceptable overall, although the convergent validity of ATT and RI should be interpreted with caution.

Confirmatory factor analysis was conducted to assess the overall fit of the measurement model. The hypothesised five-factor measurement model showed satisfactory fit: χ^2^ = 715.38, df = 289, χ^2^/df = 2.48, CFI = 0.940, TLI = 0.933, RMSEA = 0.051, and SRMR = 0.044. These results provided preliminary support for the empirical distinction among ATT, RI, HPCPI, PFPI, and FI.

Because RI and HPCPI are theoretically related, additional analyses were conducted to examine whether the two constructs were empirically distinguishable. Conceptually, RI captures the extent to which owners incorporate the human–pet relationship into their self-definition, whereas HPCPI captures their willingness to purchase clothing that matches or coordinates with their pets. Thus, RI reflects an internal identity-based psychological structure, while HPCPI reflects an externally oriented consumption intention. To further test this distinction, an alternative four-factor CFA model was estimated in which RI and HPCPI were combined into a single factor. The four-factor model showed poorer fit than the hypothesised five-factor model: χ^2^ = 1155.58, df = 293, χ^2^/df = 3.94, CFI = 0.879, TLI = 0.866, RMSEA = 0.072, and SRMR = 0.058. Compared with the four-factor alternative, the five-factor model showed significantly better fit, Δχ^2^ = 440.21, Δdf = 4, *p* < 0.001, ΔCFI = 0.061. The five-factor model also had lower AIC and BIC values than the four-factor model, AIC = 37,761.93 versus 38,194.13 and BIC = 38,143.57 versus 38,558.43. These results provide additional evidence that RI and HPCPI, although conceptually related, are not empirically redundant.

Discriminant validity was further assessed using correlation analysis and the heterotrait–monotrait ratio (HTMT). The correlation results showed significant positive associations among all core constructs, with coefficients ranging from 0.349 to 0.662, all significant at *p* < 0.001. These correlations indicate that the constructs were theoretically related and provide preliminary support for the subsequent path analyses. The HTMT values ranged from 0.437 to 0.797, all below the strict criterion of 0.85, supporting discriminant validity among the constructs. The HTMT value between HPCPI and PFPI was 0.797, also below 0.85, indicating that although the two types of purchase intention were strongly related, they did not represent the same construct. Therefore, it was appropriate to further compare the strength of the associations between RI and the two purchase intention outcomes.

Finally, potential common method bias was examined using Harman’s single-factor test and a single-factor CFA model. The unrotated principal component analysis showed that the first principal component explained 38.38% of the variance, below the commonly used 40% threshold. In addition, the single-factor CFA model showed substantially poorer fit than the five-factor model: /df = 6.78, CFI = 0.757, TLI = 0.736, RMSEA = 0.101, and SRMR = 0.078. These results suggest that common method bias was unlikely to pose a serious threat in this study.

### 4.2. Mediation Analysis

This study further examined the mediating role of relational identity in the relationships between pet attachment and the two types of pet-related fashion purchase intention. Specifically, simple mediation models were constructed following Hayes’s PROCESS Model 4. Human–pet co-fashion purchase intention and pet fashion product purchase intention were entered separately as dependent variables. Pet attachment was entered as the independent variable, and relational identity was entered as the mediator. The indirect effects were tested using the bootstrap method, with gender, age, education, and pet ownership status controlled. The results are shown in Table 4.

For human–pet co-fashion purchase intention, pet attachment was positively associated with human–pet relational identity; relational identity was positively associated with co-fashion purchase intention, and the direct association between attachment and co-fashion purchase intention remained significant after the mediator was included. The bootstrap indirect association through relational identity was also significant, supporting partial mediation (Table 4).

The same pattern was observed for pet fashion product purchase intention. Relational identity showed a significant indirect association between pet attachment and purchase intention while the direct association remained significant. Thus, partial mediation was supported for both outcomes; full coefficients and confidence intervals are reported in Table 4.

### 4.3. Comparing the Association Strengths of Pet Attachment and Human–Pet Relational Identity with the Two Purchase Intentions

To further compare the association strengths of pet attachment and human–pet relational identity with the two types of purchase intention, this study used seemingly unrelated regression (SUR) for cross-equation coefficient comparison.

Because human–pet co-fashion purchase intention and pet fashion product purchase intention were measured in the same sample and are theoretically related, estimating two regression models separately may ignore the correlation between the error terms of the two outcome equations. SUR allows the two equations to be estimated simultaneously while permitting correlated error terms. It also enables tests of whether the path coefficients of the same predictor differ significantly across dependent-variable equations. On this basis, this study compared the association strengths of pet attachment and human–pet relational identity with human–pet co-fashion purchase intention and pet fashion product purchase intention, respectively. The results are shown in Table 5.

The SUR models showed that both pet attachment and human–pet relational identity were positively associated with both purchase intentions. Cross-equation tests further indicated that the associations of both predictors were significantly stronger for human–pet co-fashion purchase intention than for general pet fashion product purchase intention. The complete coefficients and path difference tests are reported in Table 5.

### 4.4. Moderation Analysis of Fashion Involvement

**Figure 2:**
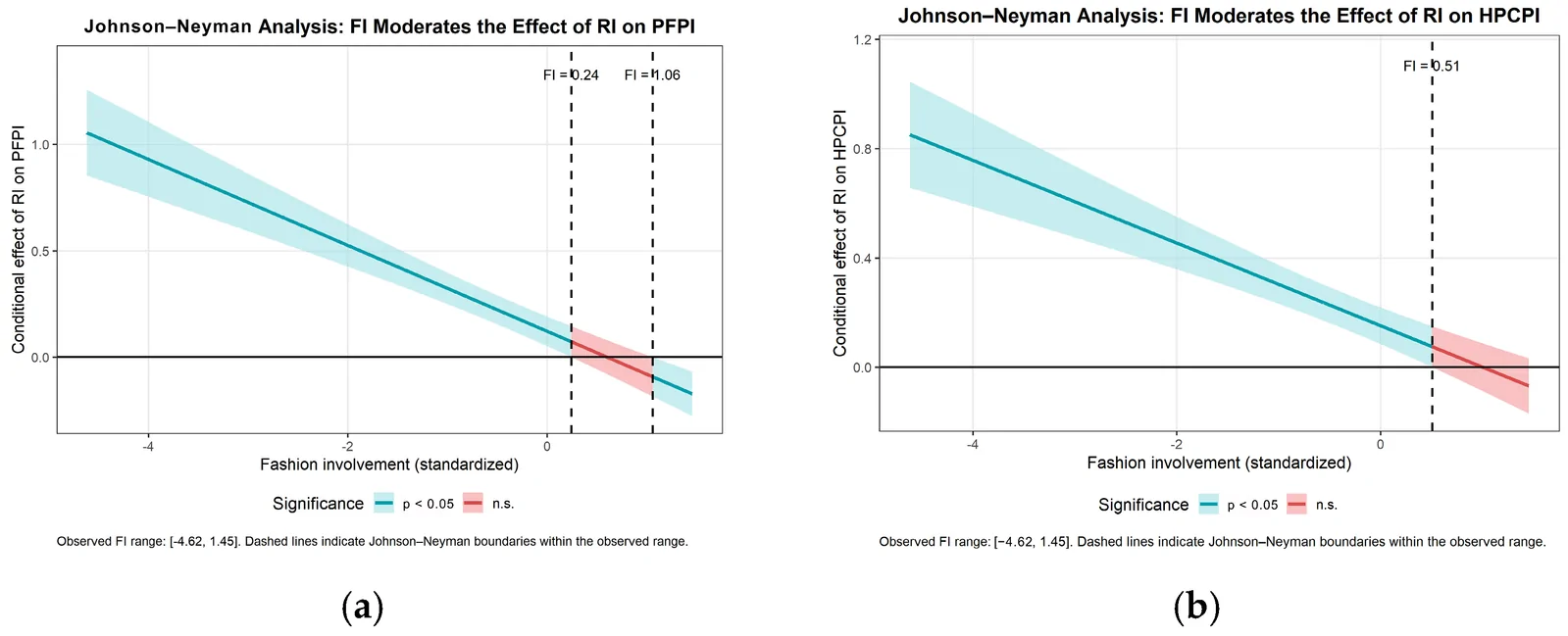
Johnson–Neyman test of the moderating effect of fashion involvement. Panel (**a**) shows the conditional effect of relational identity on human–pet co-fashion purchase intention across levels of fashion involvement. Panel (**b**) shows the conditional effect of relational identity on pet fashion product purchase intention across levels of fashion involvement.

To more precisely identify the boundary conditions under which fashion involvement changes the strength of the relationships between human–pet relational identity and the two types of purchase intention, this study used the Johnson–Neyman technique to examine the moderation effects. The results are shown in Figure 2.

In the model predicting human–pet co-fashion purchase intention, the interaction between human–pet relational identity and fashion involvement was significantly negative, B = −0.152, SE = 0.022, t = −6.960, *p* < 0.001, 95% CI = [−0.194, −0.109]. This indicates that as fashion involvement increased, the positive relationship between human–pet relational identity and human–pet co-fashion purchase intention gradually weakened. The Johnson–Neyman test showed that when standardised fashion involvement fell within the interval [0.51, 1.73], the conditional relationship between human–pet relational identity and human–pet co-fashion purchase intention was not significant. Converted into raw scale scores, this interval corresponded approximately to FI = [6.10, 7.26]. Because the theoretical range of the FI scale was 1–7 and the observed upper limit in the sample was below 7.26, the result can be interpreted within the actual sample range as follows: when fashion involvement was below approximately 6.10, human–pet relational identity was significantly and positively associated with human–pet co-fashion purchase intention; when fashion involvement exceeded approximately 6.10, this relationship was no longer significant.

In the model predicting pet fashion product purchase intention, the interaction between human–pet relational identity and fashion involvement was also significantly negative, B = −0.202, SE = 0.023, t = −8.973, *p* < 0.001, 95% CI = [−0.246, −0.158]. This indicates that fashion involvement also weakened the positive relationship between human–pet relational identity and pet fashion product purchase intention. The Johnson–Neyman test showed that when standardised fashion involvement fell within the interval [0.24, 1.06], the conditional relationship between human–pet relational identity and pet fashion product purchase intention was not significant. Converted into raw scale scores, this interval corresponded approximately to FI = [5.85, 6.63]. In other words, when fashion involvement was below approximately 5.85, human–pet relational identity was significantly and positively associated with pet fashion product purchase intention. When fashion involvement was between approximately 5.85 and 6.63, the relationship was not significant. When fashion involvement exceeded approximately 6.63, the conditional relationship showed a significant negative trend. Because this interval was located at the right tail of the sample distribution and the number of high-fashion-involvement participants was relatively limited, this negative trend should be interpreted with caution.

Overall, the interaction coefficients were negative in both models: the positive association between human–pet relational identity and purchase intention weakened as fashion involvement increased.

## 5. Discussion

### 5.1. Overall Findings and Discussion

This study examined how pet attachment, human–pet relational identity and fashion involvement jointly relate to purchase intentions for conventional pet fashion and human–pet co-fashion. Overall, pet attachment provides an important emotional basis for both forms of pet fashion consumption, while human–pet relational identity further explains how this emotional bond is associated with relationship-oriented consumption. Differences between the two purchase intentions and the moderating role of fashion involvement indicate that pet fashion consumption is shaped not only by owners’ emotional investment in their pets, but also by relational identity and the criteria used to evaluate fashion.

First, pet attachment was positively associated with both forms of purchase intention, consistent with previous evidence that pet attachment promotes pet-related consumption (Apaolaza et al., 2022; D’Souza et al., 2023). Chen et al. (2026) further found that attitudes, subjective norms, perceived behavioural control and aesthetic risk influence purchase intention for matching owner–pet clothing. Extending this work, our findings show that pet attachment is associated not only with conventional pet-apparel consumption, but also with the intention to coordinate the appearance of owner and pet. Compared with purchasing apparel solely for a pet, human–pet co-fashion requires owners to incorporate themselves into the consumption practice and may therefore be more closely connected to their emotional investment in the human–pet relationship.

Second, human–pet relational identity showed a significant indirect association between pet attachment and both forms of purchase intention. The link between pet attachment and consumption intention therefore reflects more than the owner’s regard for the pet alone. It may also involve incorporating the relationship with the pet into the owner’s self-understanding. This finding is consistent with relational-self theory, which holds that individuals can define themselves through important relationships (Cross et al., 2000, 2003). This pathway is particularly informative for human–pet co-fashion because the practice presents neither the owner nor the pet in isolation, but a relational image formed by both. Our measure captures the owner’s relational identity; however, it does not permit inferences that pets possess a corresponding identity awareness or intention for symbolic expression.

Third, human–pet relational identity was more strongly associated with purchase intention for human–pet co-fashion than with purchase intention for conventional pet fashion. This difference may reflect the distinct objects emphasised by the two consumption practices. Conventional pet fashion primarily concerns the pet’s clothing and appearance, whereas human–pet co-fashion places the owner and pet within the same visual system. Consumers who attach greater importance to their relationship with a pet may therefore be more inclined towards fashion practices that present both parties together rather than towards purchasing apparel solely for the pet.

Finally, the negative moderation by fashion involvement indicates that the association between relational identity and purchase intention varies with consumers’ level of participation in fashion. Previous research suggests that consumers with high fashion involvement generally possess greater fashion knowledge and devote more attention to clothing, styling and trend information (O’Cass, 2000, 2004). We found, however, that greater fashion involvement weakened the positive association between human–pet relational identity and both forms of purchase intention. One possible explanation is that highly fashion-involved consumers apply a broader set of independent fashion criteria rather than relying primarily on relational identity. They may give greater weight to stylistic coordination, design quality and style expression, and use richer product knowledge to distinguish among forms of coordinated dressing (Alba & Hutchinson, 1987; O’Cass, 2004). Research on authenticity similarly suggests that consumers evaluate not only the meaning conveyed by an expression, but also its credibility and sincerity (Beverland, 2005; Beverland & Farrelly, 2010).

The study did not directly measure aesthetic evaluation, need for uniqueness or authenticity judgements; therefore, this account of the moderating effect remains theoretical. A more cautious conclusion is that as consumers acquire greater fashion knowledge and evaluative resources, human–pet relational identity plays a relatively smaller role in purchase judgements. Future research could test the specific fashion-evaluation mechanisms that account for this boundary condition.

### 5.2. Theoretical Contributions

First, this study broadens the understanding of the object of consumption in research on joint human–pet consumption. Previous work has emphasised that pets are not passive objects of consumption, but participants in consumption experiences and the construction of meaning through interactions, behavioural preferences and bodily responses (Haraway, 2003, 2008; Cheetham & McEachern, 2013; Kylkilahti et al., 2016). This study primarily explains how owners and pets jointly participate in consumption. We further show that human–pet co-fashion can make the relationship between them an object of visible consumer expression. The study thus advances human–pet consumption from co-participation to relationship display and extends the unit of fashion expression from the individual-level question of “who I am” to the relational question of “who we are”.

Second, this study advances identity analysis in pet consumption from the “pet in the self” to the “relationship in the self”. Research on the extended self and related pet consumption has primarily explained how pets acquire meanings relevant to the owner’s self (Belk, 1988; Hirschman, 1994; Hill et al., 2008). Relational-self theory, by contrast, emphasises that individuals may also define themselves through relationships with significant others (Cross et al., 2000, 2003). Human–pet relational identity connects these perspectives by showing that owners may not only regard the pet as part of the self, but may also incorporate the relationship itself into their self-definition. We thereby extend relational-self theory, which has mainly been used to explain interpersonal relationships, to a cross-species consumption context and provide a new identity-based account of the link between pet attachment and relational consumption.

Third, this study refines the theoretical logic of identity-congruent consumption. Existing research has largely emphasised consumers’ tendency to choose products and consumption symbols that are congruent with their self-concepts (Sirgy, 1982; Arnould & Thompson, 2005). Our findings suggest that identity congruity may concern not only whether a consumption symbol matches the meaning of the self, but also whether the subject unit to which an identity refers corresponds to the unit represented in consumption. Human–pet relational identity refers to a “we” constituted jointly by the owner and pet, and therefore corresponds more directly to human–pet co-fashion, which presents both parties together. The moderating effect of fashion involvement further indicates that a match between identity and form of expression does not automatically translate into purchase intention, but depends on domain-specific knowledge and evaluative criteria. This study thus adds relational level and domain-specific boundary conditions to theories of identity-congruent consumption.

### 5.3. Practical Implications

This study offers practical implications for pet fashion brands in product design, marketing communication, and user operations. First, at the level of product design, brands can move from developing single pet apparel items to designing coordinated human–pet product systems. The results showed a stronger association between human–pet relational identity and human–pet co-fashion purchase intention. This suggests that some consumers do not merely want to purchase apparel for their pets. They also want to present the human–pet relationship through coordination in clothing, colour, and style. Brands can therefore develop product sets around shared owner–pet-use scenarios and create human–pet co-fashion solutions that are easier for consumers to coordinate.

Second, at the level of marketing communication, brands can adjust their communication focus according to consumers’ needs for relational expression. Brand communication should not focus only on the style, fabric, or function of pet apparel. It can also present the shared lifestyle and relational memories between owners and pets. Compared with simple product images, scenario-based content can better help consumers understand how human–pet co-fashion enters everyday life and how it functions as a form of relational expression.

Third, brands should interpret fashion involvement cautiously when segmenting consumers. The negative interaction indicates that relational identity is a weaker predictor of purchase intention at higher levels of fashion involvement, but the present data do not identify what replaces that predictive role. Brands should therefore avoid assuming that relationship appeals alone will determine responses among highly fashion-involved consumers and instead test relational storytelling alongside product-level cues such as styling coherence, design quality, perceived fashionability and occasion fit.

### 5.4. Social Implications

Human–pet co-fashion incorporates pets into systems of visual display and fashion consumption. Its social significance therefore extends beyond market growth to questions of animal welfare, the boundaries of pet anthropomorphism and sustainable consumption. Because pets are embodied participants rather than passive objects of display, the evaluation of co-fashion should consider not only its symbolic meaning for owners but also its effects on animals’ comfort, mobility, thermoregulation, stress and ability to express normal behaviour.

First, human–pet co-fashion need not be understood only as a potential welfare risk. When garments are functionally appropriate and tolerated by the animal, pet apparel can have practical welfare value in specific contexts. Recent evidence suggests that coat use can improve rest under cooler conditions, while pet wear research shows that body size, movement and fabric affect clothing pressure and comfort (Wang et al., 2026; Kim & Chae, 2026). Co-fashion may also encourage owners to pay closer attention to fit, movement, skin response and behavioural feedback when selecting garments. Its welfare value, however, depends on animal-centred design and the pet’s behavioural acceptance rather than on visual matching itself. Accordingly, the appropriateness of clothing should be evaluated in relation to the animal’s physical characteristics, environmental conditions and behavioural responses rather than assumed from human aesthetic preferences.

At the same time, human–pet co-fashion should not come at the expense of pet welfare. Poorly fitted, restrictive, heavy or insufficiently breathable garments may interfere with normal movement and heat regulation, while unfamiliar, cumbersome or excessively decorative apparel may cause stress or constrain species-typical behaviours. Owners should therefore monitor behavioural signs of discomfort or avoidance and discontinue clothing use when an animal shows difficulty moving, resting or behaving normally. Apparel and accessories should prioritise comfort, safety, freedom of movement, skin sensitivity and climate adaptability as well as adequate ventilation, thermal regulation and normal behavioural expression. Welfare requirements may also differ across species, breeds, body types and environmental conditions; therefore, a single visual or construction standard should not be assumed to be appropriate for all pets. Coordinated dressing can carry positive relational meaning, but brands and consumers should avoid reducing pets to identity-display objects or social-media content and should recognise their feelings, needs and behavioural preferences as co-living companions.

Finally, the expansion of pet fashion consumption also raises sustainability concerns. As pet apparel, accessories and coordinated human–pet product sets increase, overconsumption, infrequent use and rapid disposal may lead to resource waste. Pet fashion brands can reduce environmental burdens through durable and washable materials, modular accessories, repeated seasonal use, garment recycling and small-batch pre-sales. From a managerial perspective, product development should integrate animal-welfare criteria from the design stage rather than treating welfare as a secondary consideration after visual styling. Brands should prioritise appropriate fit, lightweight and breathable materials, skin safety, functional construction, ease of movement, thermal suitability and species-specific behavioural needs. Product information should also help consumers assess suitable usage conditions and recognise behavioural signs when a garment is not tolerated by the animal. In this sense, product development should treat the pet as a co-participant in the consumption context, with welfare and functional suitability taking priority over purely visual coordination.

### 5.5. Limitations and Future Research

This study has several limitations. First, a cross-sectional survey can identify only statistical associations among variables and cannot establish causality. The dependent variables also measured purchase intention rather than actual purchases, frequency of wear or continued use. Future research could combine experiments, longitudinal designs and behavioural data to provide further validation.

Second, the sample was recruited from a paid online platform in China. Although respondents came from different provinces and cities, the survey did not record urban–rural status or the specific type of pet owned by each participant and therefore cannot support comparisons between urban and rural consumers or among owners of different pets. Given the predominantly urban market context of pet apparel and related consumption, urban–rural differences may still affect the results. However, because the study focused on purchase intention rather than actual purchasing, practical differences such as channel availability may have had a relatively limited effect. Although the substantive context of pet fashion examined in this study is primarily associated with dogs and cats, the present data do not allow us to distinguish dog owners, cat owners, or owners of other companion animals. It therefore remains unclear whether the associations among pet attachment, relational identity, fashion involvement and purchase intention vary across pet types. Future studies could use urban–rural stratified samples and explicitly record pet type, allowing direct comparisons between dog owners, cat owners and owners of other companion animals. Such comparisons may reveal whether differences in animals’ physical characteristics, patterns of human–animal interaction and suitability for apparel lead to different forms of co-fashion consumption.

Third, this study did not distinguish among specific apparel categories, design styles or usage contexts, nor did it directly measure potential mechanisms such as perceived fashionability, aesthetic selectivity or perceived commercialisation. The present findings therefore cannot determine the specific reason for the moderating effect of fashion involvement. Future research could compare different products and usage settings and include these variables to test competing explanations.

## Figures and Tables

**Table 1:** Demographic characteristics of the sample.

Variable	Category	Frequency	Percentage
Gender	Female	304	53.81%
	Male	261	46.19%
Age	30 or below	280	49.56%
	31–40	187	33.10%
	41 or above	98	17.35%
Education	Below bachelor’s degree	57	10.09%
	Bachelor’s degree	391	69.20%
	Master’s degree or above	117	20.71%
Pet ownership status	Currently owning pets	496	87.79%
	Currently not owning pets but have prior pet ownership experience	69	12.21%

**Table 2:** Measurement items used in this study.

Variable	Code	Measurement Item	Source
Pet attachment	ATT1	I have a very close relationship with my pet.	(Archer & Ireland, 2011)
	ATT2	To me, my pet is not merely an animal, but more like an important companion.	
	ATT3	I regard my pet as an important member of my life.	
	ATT4	I treat my pet as a friend.	
	ATT5	My pet is an indispensable presence in my life.	
	ATT6	I have deep affection for my pet.	
	ATT7	I feel a special emotional bond between myself and my pet.	
Pet fashion product purchase intention	PFPI1	I am willing to purchase pet clothing.	(D’Souza et al., 2023)
	PFPI2	I would be very likely to purchase pet clothing if I had the opportunity.	
	PFPI3	Overall, I have a positive attitude toward purchasing pet clothing.	
Human–pet co-fashion purchase intention	HPCPI1	I am willing to purchase clothing that matches my pet.	(D’Souza et al., 2023)
	HPCPI2	I would be very likely to purchase clothing that coordinates with my pet if I had the opportunity.	
	HPCPI3	Overall, I am willing to purchase clothing that coordinates with my pet.	
Human–pet relational identity	RI1	The relationship between my pet and me forms part of “who I am.”	(Rast et al., 2020)
	RI2	My interaction experiences with my pet are important elements that shape “who I am.”	
	RI3	To some extent, I understand myself through the relationship between my pet and me.	
	RI4	The relationship between my pet and me forms part of the characteristics of both parties, namely myself and my pet.	
	RI5	When I think about who I am, the relationship between my pet and me is an important aspect.	
Fashion involvement	FI1	Unimportant–important	(Zaichkowsky, 1985, 1994)
	FI2	Irrelevant–relevant to me	
	FI3	Useless–useful	
	FI4(R)	Valuable–worthless	
	FI5	Uninteresting–interesting	
	FI6	Calming–exciting	
	FI7	Unattractive–attractive	
	FI8(R)	Desirable–undesirable	

**Table 3:** Reliability, validity, correlations, and HTMT results.

Construct	Cronbach’s Alpha	CR	AVE	M	SD	1	2	3	4	5
1. ATT	0.807	0.805	0.373	5.88	0.65	**0.611**				
2. RI	0.802	0.810	0.464	5.43	0.78	0.349 * /0.437	**0.681**			
3. HPCPI	0.863	0.867	0.686	5.59	1.14	0.551 * /0.662	0.492 * /0.591	**0.828**		
4. PFPI	0.797	0.802	0.577	5.87	0.88	0.467 * /0.585	0.480 * /0.604	0.662 * /0.797	**0.760**	
5. FI	0.904	0.904	0.544	5.62	0.95	0.520 * /0.608	0.493 * /0.579	0.659 * /0.743	0.647 * /0.764	**0.738**

Note: CR = composite reliability; AVE = average variance extracted. Bold diagonal values are the square roots of AVE. Values before the slash are Pearson correlation coefficients, and values after the slash are HTMT values. * *p* < 0.001. ATT = pet attachment; RI = human–pet relational identity; HPCPI = human–pet co-fashion purchase intention; PFPI = pet fashion product purchase intention; FI = fashion involvement.

**Table 4:** Mediation analysis of relational identity.

Dependent Variable	Path	B	SE	*p*	95% CI
Human–pet co-fashion purchase intention	Pet attachment → Relational identity	0.394	0.061	<0.001	[0.275, 0.513]
	Relational identity → Human–pet co-fashion purchase intention	0.523	0.097	<0.001	[0.334, 0.713]
	Direct effect	0.742	0.11	<0.001	[0.527, 0.957]
	Indirect effect	0.206	0.051	<0.001	[0.106, 0.306]
	Total effect	0.948	0.104	<0.001	[0.743, 1.152]
Pet fashion product purchase intention	Pet attachment → Relational identity	0.394	0.061	<0.001	[0.275, 0.513]
	Relational identity → Pet fashion product purchase intention	0.407	0.077	<0.001	[0.255, 0.559]
	Direct effect	0.456	0.088	<0.001	[0.285, 0.628]
	Indirect effect	0.16	0.037	<0.001	[0.087, 0.233]
	Total effect	0.617	0.089	<0.001	[0.442, 0.791]

Note: Gender, age, education, and pet ownership status were controlled. Bootstrap resampling was performed 5000 times.

**Table 5:** Comparison of path strengths for the two purchase intentions.

Predictor	Dependent Variable	B	SE	*p*
Pet attachment, ATT	Human–pet co-fashion purchase intention, HPCPI	0.742	0.061	<0.001
Pet attachment, ATT	Pet fashion product purchase intention, PFPI	0.456	0.050	<0.001
Path difference test		chi-square(1) = 24.58		<0.001
Relational identity, RI	Human–pet co-fashion purchase intention, HPCPI	0.523	0.052	<0.001
Relational identity, RI	Pet fashion product purchase intention, PFPI	0.407	0.042	<0.001
Path difference test		chi-square(1) = 5.63		0.018

Note: Gender, age, education, and pet ownership status were controlled. ATT = pet attachment; RI = human–pet relational identity; HPCPI = human–pet co-fashion purchase intention; PFPI = pet fashion product purchase intention.

## Data Availability

The data supporting the findings of this study are openly available on the Open Science Framework (OSF) at https://osf.io/4q8z2/ (accessed on 25 August 2026).
